# Association between duration of phenoxybenzamine use and postoperative delirium in suspected adrenal pheochromocytoma: a retrospective cohort study

**DOI:** 10.3389/fmed.2024.1499122

**Published:** 2024-11-06

**Authors:** Qunying Wang, Fusen Huang, Ke Wei, Jingjie Wang, Xin Zhu, Qiuju Xiong, Dan Liu

**Affiliations:** ^1^Department of Anesthesiology, The First Affiliated Hospital of Chongqing Medical University, Chongqing, China; ^2^Department of Radiology, The First Affiliated Hospital of Chongqing Medical University, Chongqing, China; ^3^Department of Urology, The First Affiliated Hospital of Chongqing Medical University, Chongqing, China

**Keywords:** phenoxybenzamine, pheochromocytoma, postoperative, delirium, adrenal

## Abstract

**Background:**

At present, the available evidence regarding the relationship between duration of phenoxybenzamine use and postoperative delirium is inadequate in suspected adrenal pheochromocytoma.

**Objective:**

To understand how changes in the duration of phenoxybenzamine use may affect postoperative delirium. The secondary objective of this study is to explore how the duration of phenoxybenzamine use may jointly influence postoperative delirium together with other interacting variables.

**Methods:**

We conducted a retrospective cohort study involving 527 participants with a preoperative diagnosis of suspected pheochromocytoma. CT characteristics, preoperative preparation, intraoperative infusion, estimated bleeding, use of intraoperative vasoactive drugs, and outcomes were obtained from all participants. Logistic regression and interaction effects were utilized to substantiate the research objectives.

**Results:**

A total of 108 (20.5%) developed postoperative delirium, which was seen in 37 (18.0%) in the pheochromocytoma group and 71 (22.0%) in the non-pheochromocytoma group. The incidence of postoperative delirium showed no statistically significant differences in the two groups. A positive association between the duration of phenoxybenzamine use and the risk of postoperative delirium was observed (OR = 1.05, 95%CI = 1.03–1.08, *p* < 0.01), independent of confounders. The relationship between the duration of phenoxybenzamine use and postoperative delirium differed according to the presence or absence of pheochromocytoma, suggesting an interactive effect (*p* < 0.05).

**Conclusion:**

This study highlights the influence of inappropriate duration of phenoxybenzamine use on the risk of incident postoperative delirium, independent of confounders. The effect of duration of phenoxybenzamine use causes a further increase in the risk of postoperative delirium, especially in non-pheochromocytomas.

## Highlights

- *Question:* There was a lack of research on whether the duration of preoperative phenoxybenzamine use leaded to postoperative delirium.
- *Findings:* Prolonged preoperative use of phenoxybenzamine increases the probability of postoperative delirium.
- *Meaning:* Preoperative medication may be altered for patients with a low likelihood of a preoperative diagnosis of pheochromocytoma.

## Introduction

Pheochromocytomas are rare neuroendocrine tumors that originate from chromaffin cells in the adrenal medulla that produce catecholamines. The primary treatment is surgical excision of the tumor. Hemodynamics, however, can alter dramatically and quickly during surgery, which significantly raises a patient’s risk of experiencing a potentially fatal complication (1). Studies have shown that preoperative alpha blockade significantly improves intraoperative hemodynamics and reduces postoperative morbidity and mortality in patients undergoing surgery for pheochromocytoma (2, 3).

At our institution, preoperative treatment with alpha blockade was a routinely required for suspected pheochromocytoma. Meanwhile, preoperative diagnosis of pheochromocytoma is not easy (4–6), which creates uncertainty about the length of preoperative preparation. Phenoxybenzamine, one of the alpha blockade agents commonly used in the preoperative treatment of pheochromocytoma, was effective in reducing the incidence of intraoperative hemodynamics instability (7, 8). According to current guidelines, patients should receive at least 7 days of preoperative alpha blockade (9). Nevertheless, the duration of Phenoxybenzamine was not entirely in line with the guidelines due to inconsistent standardization of management (10). The use of alpha blockade increased the risk of hypotension and prolonged the duration of hypotension (11, 12). The intraoperative hypotension was more harmful and was associated with increased mortality at 30 days postoperatively (13).

Postoperative delirium is one of the common postoperative complications that can prolong hospitalization and increase the financial burden on the healthcare system. Some studies had shown that intraoperative hypotension was associated with postoperative delirium in surgical patients (14, 15). Nevertheless, whether prolonged hypotension causes postoperative delirium remains controversial (16).

Prolonged administration of Phenoxybenzamine may cause an increase in the duration of intraoperative and postoperative hypotension, and it is not known whether this further contributes to an increased risk of postoperative delirium. At present, the available evidence regarding the relationship between phenoxybenzamine duration and postoperative delirium is inadequate. Based on the above studies, we hypothesized that the duration of phenoxybenzamine use may be an important indicator of postoperative delirium. This study aimed to demonstrate the correlation between phenoxybenzamine duration and postoperative delirium. The secondary objective of this study is to investigate the combined effects of phenoxybenzamine duration and interacting factors on postoperative delirium.

## Methods

### Study design, setting, and participants

The Ethics Committee of the First Affiliated Hospital of Chongqing Medical University approved permission for this prospective observational study (registration number: 2023–367), which was carried out at the hospital. Informed consent was waived by the Ethics Committee for this investigation because it was retrospective.

We reviewed the clinical information (taken from patient records) of all the patients with adrenal tumors who had surgery from July 2018 to June 2023. Suspected pheochromocytoma was considered when the CT value of adrenal tumor is >10HU on CT plain scan and the CT shows marked enhancement of the mass shadow after intravenous contrast injection; it could also be combined with positive plasma fractionated metanephrines analogues determination. Patients with a preoperative diagnosis of suspected pheochromocytoma were included, and those with a definitive preoperative diagnosis of adenoma, hypercortisolism, primary aldosteronism, ASA grade V or higher, emergency surgery, and missing data were excluded. Also, patients with a preoperative diagnosis of delirium, dementia, or mild cognitive impairment were excluded. The guidelines for Strengthening the Reporting of Observational Studies in Epidemiology were adhered to in all papers.

### Preoperative and intraoperative management

All patients included in the study were treated with alpha blockade (Phenoxybenzamine) preoperatively.

Phenoxybenzamine was started at the time of outpatient diagnosis of suspected pheochromocytoma. Then waiting for admission to the hospital for surgery, the waiting period was inconsistent for various reasons. Following admission, the dosage of phenoxybenzamine was adjusted and sustained. This was done to keep the patient’s blood pressure from rising over 130/80 mm Hg. Once alpha blockade was established, a beta blockade was added when following the achievement of alpha blockade, a beta blockade was introduced when needed to keep the heart rate (HR) below 80 beats per minute (beats/min). Fluid therapy was also administered, and some patients underwent plasma infusion.

Before anesthetic induction, all patients in the operating room had their blood pressure continuously monitored using an intra-arterial catheter. Other intraoperative monitoring included an electrocardiogram, pulse oximetry, end-expiratory carbon dioxide, nasopharyngeal temperature, urine output, and Electroencephalographic monitoring (Nacrotrend). Peripheral and central venous lines were established. All patients had general anesthesia with tracheal intubation. In general, propofol, sufentanil, and vecuronium were used to induce anesthesia, and anesthesia was maintained using propofol infusion and/or sevoflurane inhalation, as well as sufentanil and/or remifentanil. The anesthesiologist decided on the intraoperative use of vasoactive medications and/or beta-blockers. Vasodilators were often used when the systolic blood pressure (SBP) was >160 mm Hg and vasopressors when the SBP was <90 mm Hg. Phentolamine was the preferred vasodilator, with nicardipine and uradil as adjuncts. In terms of vasopressors, norepinephrine was the preferred therapy, epinephrine, phenylephrine, ephedrine, and dopamine were also employed. Esmolol was administered when the heart rate exceeded 90 beats per minute. Rehydration and blood transfusions were delivered by standard procedure. Depending on the surgeon’s preferences, the surgical procedure could be either laparoscopic or robotic-assisted; huge tumors might require open surgery, but laparoscopic to open surgery was also a possibility. Postoperatively, the patient was transferred to the post-anesthesia care unit (PACU) and, if necessary, to the intensive care unit (ICU). Patient-controlled analgesia (PCA) or multimodal analgesia was used in the postoperative period.

### Outcome measures and definition

The primary outcome was delirium within 7 days after surgery. Delirium was diagnosed using the Confusion Assessment Method (CAM). If the description in the medical record or in the nursing record matched the CAM, it was still recorded as 1 case of postoperative delirium.

The duration of phenoxybenzamine use was the duration of out-of-hospital preparation added to the duration of post-hospital administration of phenoxybenzamine. However, the duration of preoperative treatment with phenoxybenzamine was not uniform for various reasons. At the study center, surgeons tended to give phenoxybenzamine (10–15 mg, 3 times daily) for potentially longer (>7 days) in patients with a greater likelihood of pheochromocytoma (with typical symptoms, larger tumor size, and/or higher catecholamine levels). At our center, preoperative treatment with phenoxybenzamine was done out of the hospital to shorten the number of hospital days. Which had become one of the reasons for the inconsistency in the length of time phenoxybenzamine was used. Phenoxybenzamine was taken immediately when the diagnosis of suspected pheochromocytoma was made, and phenoxybenzamine was discontinued when catecholamine results ruled out pheochromocytoma. In patients with atypical symptoms who were also not tested for catecholamines, the duration of treatment may be shorter and variable. It depends on the experience of the physician. Phenoxybenzamine dosage statistics were the combination of daily usage.

The diagnosis of pheochromocytoma was determined by postoperative pathology. Suspicious pheochromocytoma was considered in patients with paroxysmal hypertension; typical triad of headache, sweating, and tachycardia; or adrenal incidentalomas with CT plains suggestive of lack of lipids and a CT value >10 HU.

Intraoperative hypotensive was defined as at least one episode of mean arterial pressure below 55 mmHg (17, 18). Intraoperative hypotension was usually treated with fluids, norepinephrine, epinephrine, dopamine, phenylephrine, or ephedrine. The need for blood transfusion depends on the amount of hemoglobin and the experience of the anesthesiologist.

### Collection of clinical characteristics

By reviewing the patient’s medical records during hospitalization, clinical characteristics were collected, including age, weight, height, ASA classification, type of adrenal tumor, and preoperative comorbidities; and perioperative indicators, including anesthetic drugs (midazolam, sufentanil, vecuronium, etc), type of surgery, length of anesthesia, length of surgery, estimated intraoperative blood loss, fluid therapy, the incidence of admission to the intensive care unit (ICU), postoperative complications, and length of hospital stay. In addition, intraoperative use of antihypertensive and antihypertensive drugs was evaluated. The worst pain score over the first postoperative 7 days was also collected.

Tumor size and enhanced versus unenhanced CT values were measured by reviewing patients’ adrenal CT images completed by an attending radiologist with more than 5 years of experience.

### Statistical analyses

Histogram distribution was used to determine whether variables were normally distributed. All normally distributed continuous variables were expressed presented as mean ± SD, and skewed continuous variables were described as median [interquartile range (IQR)]. Categorical variables were presented as frequencies (%). Comparison of continuous variables among groups was performed with the use of the independent samples Student’s *t*-test or Mann–Whitney *U*-test depending on the normality of the distribution, and categorical data were compared by chi-square or Fisher’s exact test as appropriate.

The effect of days of phenoxybenzamine use on postoperative delirium was evaluated using binary logistic regression models [odd ratio (OR) and 95% confidence interval (CI)] with adjustment for major covariables. We selected these confounders based on judgment, previous scientific literature, all significant covariates in the univariate analysis, or their associations with the outcomes of interest or a change in effect estimate of more than 10%. We constructed 2 models: Model 1 adjusted for age, sex, and BMI. Model 2 was additionally adjusted for Preoperative comorbidities, CT characteristics, types of surgery, duration of surgery, estimated bleeding, and transfusions.

Subgroup analysis examined the relationship between Days of Phenoxybenzamine use and postoperative delirium according to Subgroup variables, especially between the pheochromocytoma group and the non-pheochromocytoma group. Interaction across subgroups was tested using the likelihood ratio test. Missing data accounted for <5% of the data set and were handled by list-wise deletion on an analysis basis. All analyses were performed using R Statistical Software (Version 4.2.2,^1^ The R Foundation) and Free Statistics analysis platform (Version 1.9, Beijing, China).^2^ A two-sided *p* < 0.05 was considered statistically significant.

## Results

**Figure 1:**
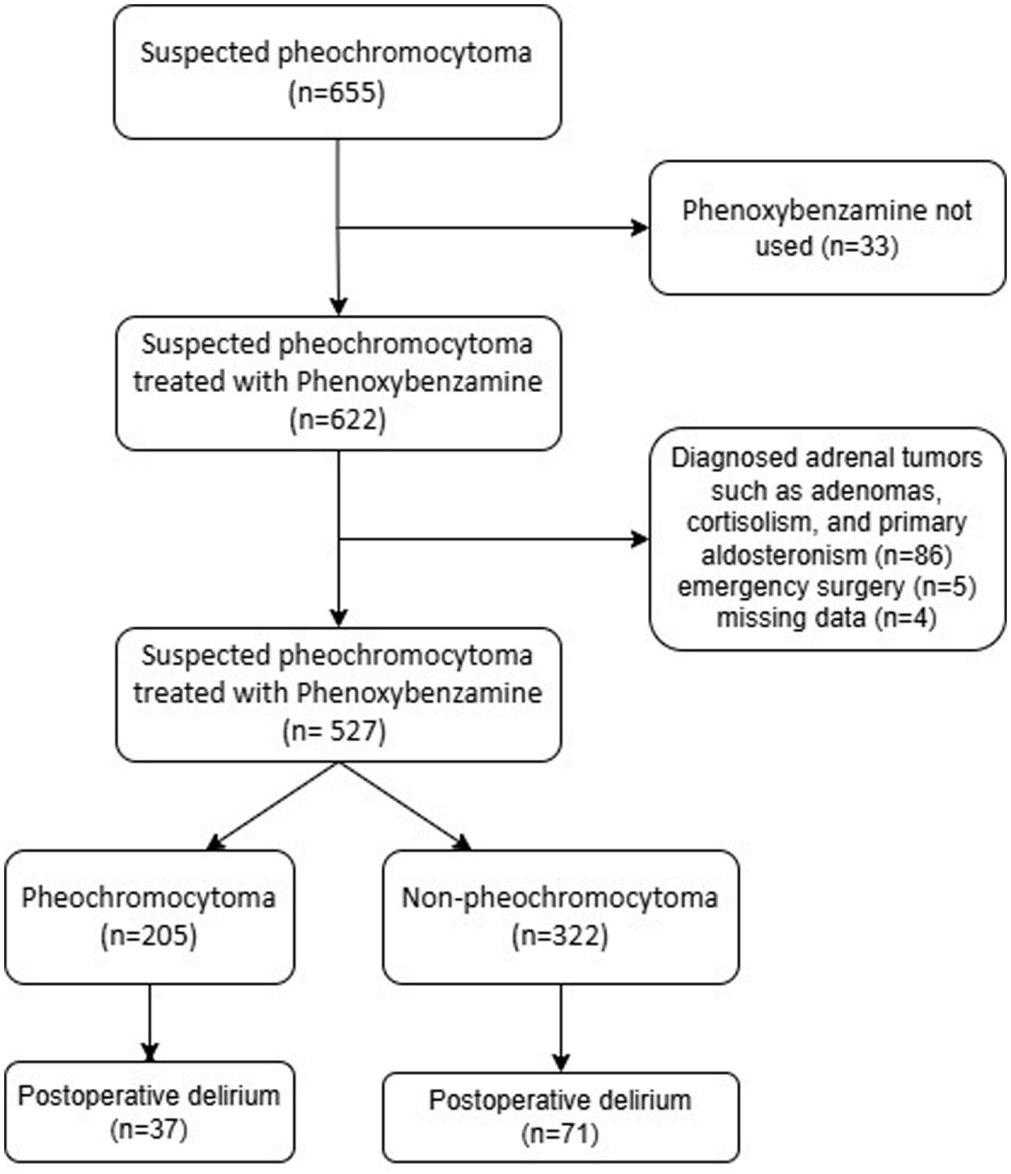
The flow chart of the study.

A total of 527 patients were included after strict screening according to the inclusion and exclusion criteria. Of these, 205 cases (38.9%) were definitively diagnosed as pheochromocytoma (Figure 1). A total of 108 (20.5%) developed postoperative delirium, which was seen in 37 (18.0%) in the pheochromocytoma group and 71 (22.0%) in the non-pheochromocytoma group. Table 1 shows the baseline characteristics of the subjects included in the final analysis. Table 2 shows the differences in perioperative correlates between the pheochromocytoma group and the non-pheochromocytoma group.

**Table 1:** Patient demographic information.

Variables	Total (*n* = 527)	Non-pheochromocytoma (*n* = 322)	Pheochromocytoma (*n* = 205)	*p*
Age (y), Mean ± SD	52.8 ± 12.7	53.3 ± 12.4	51.9 ± 13.2	0.206
Height (cm), Mean ± SD	160.6 ± 11.1	160.9 ± 9.3	160.3 ± 13.4	0.537
Weight (Kg), Mean ± SD	62.6 ± 12.5	63.9 ± 12.0	60.6 ± 12.9	0.003
BMI (Kg m^−2^), Mean ± SD	26.7 ± 40.1	25.4 ± 20.6	28.7 ± 58.9	0.358
ASA, *n* (%)				< 0.001
I	2 (0.4)	2 (0.6)	0 (0)	
II	197 (37.4)	170 (52.8)	27 (13.2)	
III	317 (60.2)	149 (46.3)	168 (82)	
IV	11 (2.1)	1 (0.3)	10 (4.9)	
Type of diseases, *n* (%)				< 0.001
Pheochromocytoma	205 (38.9)	0 (0)	205 (100)	
Adenoma	277 (52.6)	277 (85.7)	0 (0)	
Hyperaldosteronism	6 (1.1)	6 (1.9)	0 (0)	
Cortisolism	21 (4.0)	21 (6.5)	0 (0)	
Cyst	8 (1.5)	8 (2.5)	0 (0)	
Hemangioma	9 (1.7)	9 (2.8)	0 (0)	
Teratoma	1 (0.2)	1 (0.3)	0 (0)	
Hypertension, *n* (%)	307 (58.3)	176 (54.7)	131 (63.9)	0.036
DM, *n* (%)	112 (21.3)	54 (16.8)	58 (28.3)	0.002
Dislipidemia, *n* (%)	45 (8.5)	30 (9.3)	15 (7.3)	0.423
Cerebral infarction, *n* (%)	18 (3.4)	15 (4.7)	3 (1.5)	0.049
Other combidity, *n* (%)	145 (27.5)	87 (27)	58 (28.3)	0.75
CT diameter (cm), Median (IQR)	3.9 (2.6, 5.5)	3.0 (2.3, 4.1)	5.1 (4.3, 6.3)	< 0.001
CT unenhanced, Median (IQR)	25.0 (14.0, 38.5)	16.0 (9.0, 24.0)	39.0 (35.0, 43.0)	< 0.001

BMI, body mass index; ASA, American Society of Anesthesiologists; DM, Diabetes mellitus; CT, computerized tomography.

**Table 2:** Perioperative information of patient.

Variables	Total (*n* = 527)	Non-pheochromocytoma (*n* = 322)	Pheochromocytoma (*n* = 205)	*p*
Preoperative treatment				
Phenoxybenzamine duration (day), Median (IQR)	7.0 (4.0, 11.0)	6.0 (4.0, 8.0)	9.0 (5.0, 17.0)	< 0.001
Phenoxybenzamine (mg), Median (IQR)	290.0 (150.0, 625.0)	210.0 (112.5, 360.0)	480.0 (270.0, 1070.0)	< 0.001
RBC transfusion, *n* (%)	44 (8.3)	13 (4)	31 (15.1)	< 0.001
Plasma transfusion, *n* (%)	27 (5.1)	7 (2.2)	20 (9.8)	< 0.001
Intraoperative information and treatment				
Surgery type, *n* (%)				< 0.001
Laparoscopic	401 (76.1)	270 (83.9)	131 (63.9)	
Robotic-assisted	103 (19.5)	40 (12.4)	63 (30.7)	
Open surgery	20 (3.8)	9 (2.8)	11 (5.4)	
Intermediate open	3 (0.6)	3 (0.9)	0 (0)	
Midazolam, *n* (%)	455 (86.3)	318 (98.8)	137 (66.8)	< 0.001
Sufentanil (ug), Median (IQR)	40.0 (35.0, 45.0)	40.0 (35.0, 45.0)	40.0 (35.0, 45.0)	0.661
Remifentanil (mg), Median (IQR)	1.2 (1.0, 1.7)	1.2 (0.9, 1.4)	1.5 (1.2, 2.0)	< 0.001
Vecuronium (mg), Median (IQR)	10.0 (9.0, 11.7)	9.7 (8.5, 10.7)	11.0 (9.7, 12.8)	< 0.001
Introperative hypotension, *n* (%)	96 (18.2)	49 (15.2)	47 (22.9)	0.025
Phenylephrine (ug) Median (IQR)	200.0 (150.0, 250.0)	250.0 (200.0, 300.0)	100.0 (100.0, 150.0)	< 0.001
Norepinephrine, *n* (%)	394 (74.8)	223 (69.3)	171 (83.4)	< 0.001
Epinephrine, *n* (%)	27 (5.1)	3 (0.9)	24 (11.7)	< 0.001
Phentolamine, *n* (%)	68 (12.9)	0 (0)	68 (33.2)	< 0.001
Dopamine, *n* (%)	22 (4.2)	10 (3.1)	12 (5.9)	0.124
Esmolol, *n* (%)	57 (10.8)	1 (0.3)	56 (27.3)	< 0.001
RBC transfusion, *n* (%)	24 (4.6)	7 (2.2)	17 (8.3)	0.001
Plasma transfusion, n (%)	14 (2.7)	5 (1.6)	9 (4.4)	0.048
Anesthesia duration (min), Median (IQR)	150.0 (120.0, 200.0)	140.0 (105.0, 170.0)	180.0 (140.0, 235.0)	< 0.001
Surgery duration (min), Median (IQR)	110.0 (80.0, 155.0)	100.0 (73.5, 135.0)	130.0 (95.0, 190.0)	< 0.001
Estimate blood loss (ml), Median (IQR)	200.0 (100.0, 300.0)	200.0 (100.0, 300.0)	200.0 (100.0, 350.0)	0.017
Urine volume (ml), Median (IQR)	200.0 (100.0, 400.0)	200.0 (100.0, 300.0)	300.0 (150.0, 500.0)	< 0.001
Postoperative information				
Neostigmine (mg), Median (IQR)	1.0 (1.0, 2.0)	1.0 (1.0, 2.0)	1.0 (1.0, 2.0)	0.014
PACU duration (min), Median (IQR)	70.0 (60.0, 90.0)	65.0 (60.0, 80.0)	80.0 (60.0, 100.0)	< 0.001
ICU, *n* (%)	11 (2.1)	2 (0.6)	9 (4.4)	0.004
Postoperative delirium, *n* (%)	108 (20.5)	71 (22)	37 (18)	0.267
Infection, *n* (%)	10 (1.9)	4 (1.2)	6 (2.9)	0.198
Thrombsis, *n* (%)	15 (2.8)	7 (2.2)	8 (3.9)	0.245
Adrenal crisis, *n* (%)	2 (0.4)	2 (0.6)	0 (0)	0.524
Postoperative NRS _max_, Median (IQR)	2.0 (1.0, 3.0)	2.0 (1.0, 3.0)	3.0 (2.0, 4.0)	< 0.001
Hospital duration (day), Median (IQR)	5.0 (4.0, 6.0)	4.0 (3.0, 5.0)	5.0 (4.0, 7.0)	< 0.001

RBC, Red blood cell; PACU, post-anesthesia care unit; ICU, intensive care unit; NRS, numerical rating scale.

**Figure 2:**
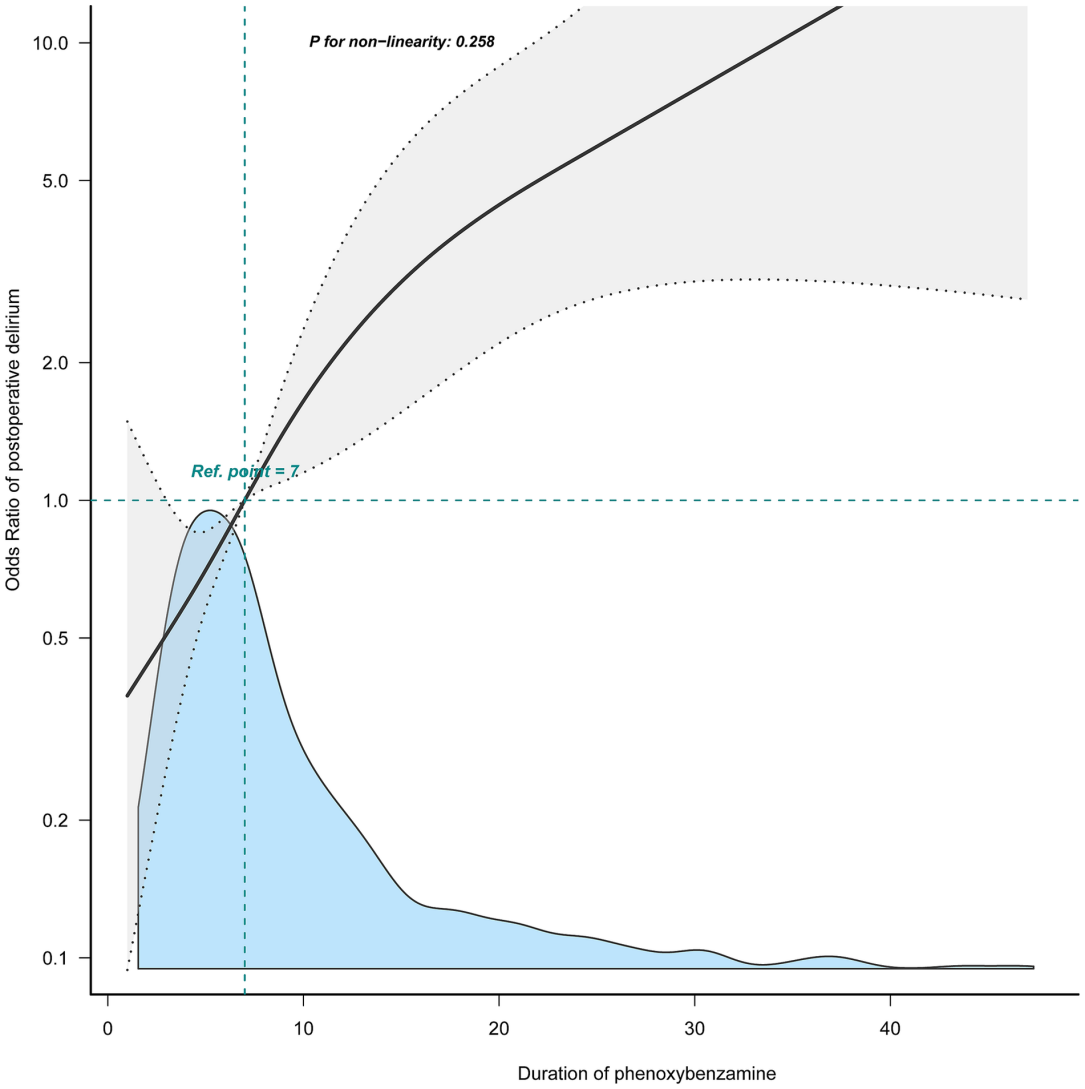
The relationship between the duration of phenoxybenzamine use and the risk of postoperative delirium.

Figure 2 shows a linear relationship between the duration of phenoxybenzamine use and the risk of postoperative delirium. In univariate logistic regression analyses, the duration of phenoxybenzamine use was expressed as a continuous variable (Per 1 day was positively associated with the probability of postoperative delirium) (OR = 1.04, 95% CI = 1.02–1.06, *p* < 0.001). This association was stable and remained statistically significant (OR = 1.05, 95% CI = 1.03–1.08, *p* < 0.001), independent of potential confounders (Table 3, model 2).

**Table 3:** Multivariate analysis of postoperative delirium.

Outcome	Non-adjusted model		Model I		Model II	
	OR (95% CI)	*P*	OR (95% CI)	*P*	OR (95% CI)	*P*
Duration of phenoxybenzamine	1.04 (1.02–1.06)	<0.001	1.06 (1.03–1.08)	<0.001	1.05 (1.03–1.08)	<0.001

Model I: Adjust for sex, age, and BMI; Model II: Adjust for Model I + ASA, Cerebral infarction, Midazolam, Sufentanil, neostigmine, Anesthesia duration, estimated blood loss, and postoperative VAS max.

**Figure 3:**
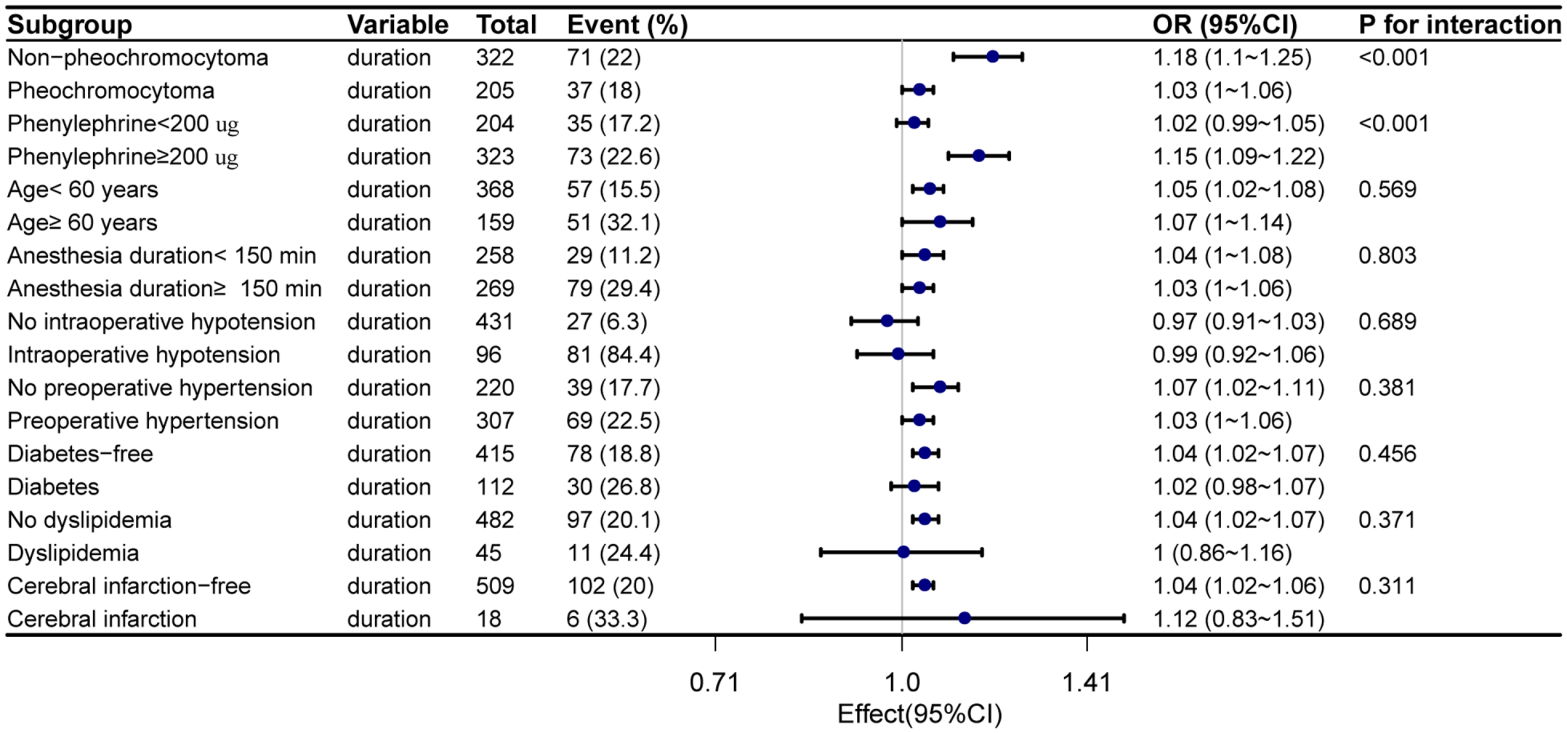
Relationship between the duration of preoperative phenoxybenzamine administration and postoperative delirium in subgroup. Adjust for Sex, BMI, ASA, neostigmine, postoperative NRS _max,_ Midazolam, Sufentanil, and estimated blood loss.

We also performed stratified analyses according to pheochromocytoma and non-Pheochromocytoma. We found that the relationship between the duration of phenoxybenzamine use and postoperative delirium was statistically significant across the subgroups (the *p*-value for the interaction likelihood ratio test was *p* < 0.001). In the non-pheochromocytoma group, each additional day of preoperative phenoxybenzamine use was associated with an 18% increase in the probability of postoperative delirium (OR = 1.18, 95% CI = 1.1–1.25, *p* < 0.001) (Figure 3).

## Discussion

In these patients with a preoperative diagnosis of suspected pheochromocytoma retrospective cohort study, we first demonstrated that the duration of phenoxybenzamine use was independently associated with an increase in the risk of developing postoperative delirium. It is worth mentioning that an interaction was also observed between the duration of phenoxybenzamine and pheochromocytoma and non-pheochromocytoma in their influence on postoperative delirium (*p* < 0.05).

Our study observed positive associations of duration of phenoxybenzamine use with postoperative delirium in the context of patients with preoperative diagnosis of suspected pheochromocytoma, especially in the non-pheochromocytoma subgroup. Inadequate cerebral perfusion and impaired oxygenation were considered the most important contributing factors to postoperative delirium (14, 15). The occurrence of postoperative delirium may be related to intraoperative and postoperative hypotension. Kim (19) team found that alpha blockade was a predictor of intraoperative hemodynamic instability. The choice to treat normotensive pheochromocytomas with phenoxybenzamine before surgery may result in dangerously low blood pressure (11). According to Groeben et al. (20), receiving alpha blockade medication before surgery increased the need for vasopressors and intraoperative hypotension (21). Not singly but in pairs, Groeben et al. (20) similarly questioned the role of preoperative alpha blockade in preventing intraoperative hypertension, and preoperative use of alpha blockade may be associated with a higher incidence of complications related to hemodynamic instability (20). Our findings further confirm the higher risk of postoperative delirium in the intraoperative hypotensive population.

Furthermore, we noticed variations in the relationships between the various durations of phenoxybenzamine use and postoperative delirium in strata with pheochromocytoma and those without. The risk of postoperative delirium was greater in the non-pheochromocytoma group. This may be explained by the fact that these biochemically silent “phaeochromocytomas” were not true pheochromocytomas, did not secrete catecholamines, and did not constrict blood vessels throughout the body. Alpha-blocker use may result in alterations to vascular tone response (19). In the non-pheochromocytoma group, Hypotension lasted longer. This type of vessel wall responds more poorly to vasoconstrictors and requires higher doses of vasoconstrictors to maintain blood pressure, including phenylephrine. A study suggests that phenylephrine impairs cerebral perfusion by constricting cerebral blood vessels leading to an increased risk of postoperative delirium (16). This was confirmed in our study, where a greater amount of phenylephrine was used in the non pheochromocytoma group, with an increased risk of postoperative delirium.

However, for non-pheochromocytomas that cannot be definitively diagnosed preoperatively, the use of alpha-blockers was still necessary if these procedures were not undertaken in specialized adrenal centers (22). For the preparation of such patients, short-acting alpha-blockers are more appropriate than phenoxybenzamine (23). Other medications such as calcium channel blockers may also provide options (24). Possibly similar to the mechanism in dopamine-only pheochromocytomas, a shorter preoperative preparation time has been suggested (23). Therefore, preoperative preparation should be individualized for this group of patients (23). For patients with a low likelihood of pheochromocytoma, the duration of preoperative preparation should be minimized by using short-acting drugs or other drug alternatives. For institutions with specialized adrenal centers, there are more possibilities for preoperative medication choices (22).

This is the first time that the latest summary-level data have been applied to explore the connections between the duration of phenoxybenzamine use and postoperative delirium. The research perspective taken in this study was genuine, and it had excellent novelty and therapeutic application value. The current study provides robust evidence of the relationship between the duration of phenoxybenzamine use and the risk of postoperative delirium in patients with suspected pheochromocytoma through a thorough consideration of potential sources of confounding and biases. Nevertheless, several limitations merit consideration. First, as an observational study, we cannot establish a causal relationship between the duration of phenoxybenzamine use and the risk of postoperative delirium in the suspected pheochromocytoma population. The sample size in this study was relatively small, which limited the statistical power and exploration of interactions. The results should be interpreted with caution. Our basis for determining postoperative delirium is not a universal standard. We considered patients who matched the description of CAM in the medical record or the nursing record as postoperative delirium, which may have resulted in more cases of delirium than the actual number of cases. Also, there may be unmeasured selection bias with confounding factors due to incomplete information on some patients in the database, our exclusion of some patients, and the inability of the database to provide additional baseline data, such as Catecholamine secretion levels.

## Conclusion

This cohort study found that positive relationship between the number of days of phenoxybenzamine use and postoperative delirium in suspected pheochromocytomas. Furthermore, this relationship is further enhanced in the non-pheochromocytoma subgroup. The findings of this study require further validation and confirmation.

## Data Availability

The original contributions presented in the study are included in the article/supplementary material, further inquiries can be directed to the corresponding author.

## Footnotes

^1^ https://www.R-project.org

^2^ https://www.clinicalscientists.cn/freestatistics
